# Monerdaktar: A large online mental health service to improve access to care in bangladesh during the COVID-19 pandemic

**DOI:** 10.1192/j.eurpsy.2021.134

**Published:** 2021-08-13

**Authors:** T. Rashid Soron, Z.F. Chowdhury

**Affiliations:** Compaince And Finance, Telepsychiatry Research and Innovation Netowrk Ltd, Dhaka, Bangladesh

**Keywords:** digital health, mental health, telepsychiatry

## Abstract

**Background:**

More than 92% of people in Bangladesh are deprived from any sort of mental health care due to severe scarcity of mental health professionals, widespread stigma, lack of awareness, the inability to travel from remote area to Dhaka and maintaining the cost of travel and clinics. Moreover, the COVID-19 crisis made the scenario worse. To solve this problem we designed, developed and implemented “Monerdaktar”.

**Methods:**

The process development Monerdaktar- website and mobile application started with the initial idea and concept by TRS followed by extensive literature review and naturalistic observation of the mental health care service delivery from two tertiary hospitals in Bangladesh. We conducted 3 focus group discussion with the patients, their care givers, mental health professions. Based on the user feedback and technical suggestion of the mental health professional and IT professionals we developed the prototype of the Monerdaktar mobile application and website. After piloting for two months, the final version of the mobile application and website was finalized incorporating the feedback of the patients and experts.

**Result:**

Monerdaktar created the unique opportunity to connect with the most the reputed mental health professional both psychiatrists and clinical psychologists online. Moreover, monerdaktar delivered the service free of cost to more than 700 clients during the peak of COVID crisis in Bangladesh.

**Conclusion:**

The COVID-19 crisis has potentiated the acceptance and adaptation of the Moenrdaktar solved the long-standing crisis of access to mental health care in Bangladesh and ensure the evidence-based care from anywhere.

**Disclosure:**

The Monerdaktar website and mobile application was design and developed by under the leadership of Dr. Tanjir Rashid Soron. Though the initial support was delivered free of cost, the consulting expert psychiatrist and Clinical Psychologist may take their

